# Comparison of anterior corneal aberrations measured by Scheimpflug and Placido Disc System for myopes

**DOI:** 10.1186/s12886-022-02753-9

**Published:** 2022-12-28

**Authors:** Wei Lou, Wei Du, Haiying Jin, Ying Hu

**Affiliations:** 1 Department of Ophthalmology, Shanghai East Hospital, Tongji University School of Medicine, No.150 Jimo Road, Shanghai, 200120 China; 2 Department of Ophthalmology, Shanghai Sixth People’s Hospital, Shanghai Jiao Tong University, No.600 Yishan Road, Shanghai, 200030 China

**Keywords:** Agreement, Pentacam, KR-1W, Aberration, Myopia

## Abstract

**Background:**

To ascertain the agreement of corneal aberrations obtained from the Pentacam and the KR-1W in myopic populations and to investigate the influence of the level of myopia as well as the laterality on the agreement.

**Methods:**

In this observational study, a rotating Scheimpflug camera (Pentacam AXL) and a Hartmann-Shack wavefront analyzer with Placido-disc topographer (KR-1W) were used to measure the aberrations of myopes in the anterior corneal surface by one experienced operator. All examinations were computed across a 6 mm diameter. Six subgroups were generated according to the degree of myopia (mild, moderate, and severe myopia) and the laterality of eyes (right and left eyes).

**Results:**

The study included 245 eyes of 170 participants. For certain anterior corneal aberrations, statistically significant differences existed between the Pentacam and the KR-1W (all *P* < .05). The values of Zernike (Z)(2,0), Z(2,2), Z(3,1), and Z(4,0) varied in all levels of myopia regardless of the laterality, with the values of the Pentacam constantly larger than the KR-1W in the measurement of Z(2,0), Z(2,2), and Z(4,0). For 2nd to 6th aberrations, both instruments correlated poorly to moderately. The width of limits of agreement between the two instruments was clinically too wide (> 0.1 μm) for aberrations closely correlated with visual quality, including Z(3, ± 3), Z(3, ± 1), and Z(4,0), and almost all aberrations, indicating poor agreement.

**Conclusions:**

In clinical practice, the Pentacam based on Scheimpflug technology and the KR-1W based on Placido Disc System are not interchangeable in measuring anterior corneal aberration for myopes regardless of myopia degree and the laterality, suggesting that a consistent instrument should be selected for surgical design as well as follow-up.

**Supplementary Information:**

The online version contains supplementary material available at 10.1186/s12886-022-02753-9.

## Background

Cataract surgery and corneal refractive surgery are developing towards personalized refractive surgery. While improving visual acuity, increasing attention is being placed on the role of aberrations in visual quality [1–3]. The total ocular aberrations consist of corneal and intraocular parts [4]. Aberrations can be further divided into lower-order and higher-order aberrations (HOAs) [5]. A recent study has shown that corneal HOAs can be a sensitive parameter to differentiate early keratoconus from normal eyes [6]. Furthermore, corneal aberrations have been widely used to evaluate dry eye and orthokeratology and guide the treatment strategies for cataract and refractive surgery [7–9].

Currently, devices based on Placido-disc, Hartmann-Shack, ray tracing, or Scheimpflug are routinely used in clinical aberration measurement, of which Pentacam and KR-1W are two common instruments [10–12]. The former can only measure corneal aberration (including anterior and posterior corneal surface) based on Scheimpflug technology. In contrast, the latter can measure various ocular aberrations (including corneal, internal, and total aberrations). Regarding the corneal aberrations, the KR-1W can only be used to assess aberrations in the anterior corneal surface because of a Placido-disc nature.

Previous studies have compared the differences between Pentacam, Galilei, OPD scan, iDesign, iTrace system, and KR-1W in the measurement of aberrations [13–15]. However, to the best of our knowledge, there is few research investigating the differences between Pentacam and KR-1W in measuring corneal aberrations of myopes. Notably, corneal aberration measurements of candidates for refractive surgery are one of the most widely used scenarios for these technologies. A better understanding of the agreement of device measurements can provide clinicians with useful information for measuring, correcting, and monitoring aberrations around refractive and cataract surgery. We, therefore, aimed to study the agreement between the two instruments in anterior corneal aberration measurement of myopes and further analyze the effects of different myopia levels and laterality on the agreements.

## Methods

### Study participants

The cross-sectional observational study achieved the approval of local ethics institution. All procedures strictly adhered to the tenets of the Declaration of Helsinki, with written informed consent obtained from all participants. All subjects were refractive surgery candidates from the Department of Ophthalmology, Shanghai Sixth People's Hospital, between October 2018 and December 2020. Before the recruitment, comprehensive eye examinations were conducted, including complete ophthalmoscopic examination, intraocular pressure measurement using a non-contact tonometer, and ultra-wide-field retinal imaging. Inclusion criteria were healthy myopic subjects, ≥ 18 years old, with a best corrected visual acuity (BCVA) of 20/20 or more. Refractive error was measured by an autorefractometer (NIDEK AOS-1500, Japan) with cycloplegia, of which the sphere > -0.5 diopters (D) and the cylinder ≤ -1.5 D were accepted. The exclusion criteria were irregular astigmatism, ocular diseases, e.g., dry eyes, corneal diseases, glaucoma, uveitis, ocular trauma, ocular surgery history, contact lens wear within the previous 2 weeks, or rigid gas permeable lens wear within 4 weeks. Those with opacities in the media that seriously affect transparency, such as corneal leucoma, cataracts, and vitreous opacity, were also excluded.

To study the differences in aberration results measured by the two instruments under different refractive states, we created three groups according to the sphere: mild (-0,5D - -3.0D), moderate (-3.25D - -6.0D), and severe (≥ -6.25D). Similarly, for a more detailed comparison, we further divided each group mentioned above into two separate subgroups (right eye group and left eye group) according to the laterality of eyes. Thus, six studied subgroups (subgroup 1 – subgroup 6) were generated corresponding to mild, moderate, and severe myopia in the right and left eye groups, respectively.

### Instruments and procedures

All the enrolled eyes were examined on a rotating Scheimpflug camera (Pentacam AXL, Oculus Optikgerate GmbH, Germany) and a Hartmann-Shack wavefront sensor (KR-1W; Topcon Corporation, Tokyo, Japan). If the bilateral eyes of the volunteer were included, both eyes were analyzed. The measurements were carried out in a dark room without cycloplegia by one experienced operator. If the measurements did not meet the standard quality control of the instruments, the operator was allowed to examine the participants once more until accurate readings were obtained. Before starting both examinations, the volunteers were asked to blink properly to reduce the errors caused by the tear film.

The Pentacam is a Scheimpflug-based instrument using a rotating camera. It uses a blue diode laser as the light source to obtain three-dimensional scans of the anterior segment of the eye. It then performs a Zernike polynomial to acquire the wavefront aberrations on the anterior and posterior surfaces of the cornea. For the measurements of the Pentacam, the participants took a sitting position, placed the lower jaw on the mandibular rest of the instrument, and looked at the fixation target in the blue light target in the center of the rotation axis with both eyes open. The operator used the lever to focus the machine accurately in the direction displayed on the screen, and the Pentacam automatically started the measurement after completing the alignment. The 25-image mode was chosen so that the rotating camera could acquire 25 scans within seconds.

The KR-1W is an aberrometer combining Placido-disc topography and Hartmann-Shack wavefront sensor. It uses conventional Placido-disc technology to evaluate corneal curvature within a range from 5.00 mm to 10.00 mm (in 0.01 mm increments). There are 38 Placido rings in the topographer, which can assess 13,680 data points. The anterior corneal HOAs are calculated using collected topography data. The automatic mode of the KR-1W instrument was used. In this mode, the device can center, focus and measure after the operator presses the button on the operating lever.

For Pentacam examinations, we selected “WFA cornea front” from 5 available choices in the “Zernike display” because the KR-1W measures the corneal aberrations using a Placido-disc topographer, and this measures only the anterior surface of the cornea. Twenty-five parameters, computed across a 6 mm diameter, were used for the analysis: the individual Zernike coefficients from the second (Z(2,m)) to the sixth order (Z(6,m)), Z is the angular frequency.

The precision of aberration measurements using these two devices has been extensively reported and therefore has not been evaluated in this study [16–20].

### Statistical analysis

SPSS software (version 25, SPSS Inc., Chicago, IL, U.S.) and Prism (version 8.0.1, GraphPad Software, San Diego, CA) were used for statistical analyses. Statistical significance was defined as *P* < 0.05. All aberrations were displayed as mean ± standard deviation (SD). Paired t-tests or Wilcoxon two-related-samples tests was used to compare corneal aberrations measured by the Pentacam and the KR-1W, according to the normality, which was checked using the Kolmogorov–Smirnov test. Also, to measure the correlations between measurements of the two devices, the correlation coefficient Pearson’s R or Spearman’s R was also used, where R values smaller than 0.4 indicated low correlation, between 0.4 and 0.8 represented moderate correlation, and greater than 0.8 denoted high correlation. Bland–Altman analysis was conducted to investigate the agreement of all aberrations measured from the two instruments. The mean difference (bias) and 95% LoA were calculated. Additionally, Bland–Altman graphs were further constructed to visualize the agreement of various most important vision quality related parameters (Z(4,0), Z(3, ± 1), Z(3, ± 3)) achieved from the two devices.

## Results

### Baseline characteristics of study subjects

In our study, 245 eyes (120 right eyes and 125 left eyes) from 170 participants (93 women and 77 men) were analyzed. The mean age of study subjects was 26.11 ± 6.38 years, ranging from 18 to 40 years. The mean sphere was -4.30 ± 2.00 D. Table 1 lists the demographic information of the participants in the three myopia subgroups.

**Table 1:** Demographics of the study population

Parameter	Mild myopia group	Moderate myopia group	Severe myopia group
Eye (N)	88	91	66
OD/OS (N)	38 / 50	45 / 46	37 / 29
Age (Y)			
Mean ± SD	24.99 ± 6.15	25.82 ± 5.89	28.41 ± 6.46
Sphere (D)			
Mean ± SD	-2.26 ± 0.61	-4.32 ± 0.86	-6.90 ± 0.74
Spherical Equivalent (D)			
Mean ± SD	-2.56 ± 0.64	-4.64 ± 0.88	-7.31 ± 0.75

*D* Diopter, *N* Number, *Y* Year, *SD* Standard deviation

### The comparison of aberrations between the two devices

The measured parameters from the two instruments were compared using appropriate statistical methods (Paired t-tests or Wilcoxon two-related-samples tests) according to the normality. Supplemental Table 1 shows a significant difference between Pentacam and KR-1W for a portion of the aberration measurements. Specifically, regardless of myopic levels or laterality, the values of Z(2,0) (defocus), Z(2,2) (axial astigmatism), Z(3,1) (horizontal coma) and Z(4,0) (primary spherical aberration, SA) differed (Table 2). Furthermore, for the measurement of Z(2,0), Z(2,2), and Z(4,0) in all subgroups, the results of Pentacam were constantly larger than KR-1W (notably, because Z(4,0) does not conform to the positive distribution in subgroup 4, it is necessary to compare the value by the median (quartile): Pentacam: (0.233 (0.080)), KR-1W: (0.205 (0.056)). Additionally, for the same myopic level, the difference in individual aberration measurements between the two devices in the 2nd to 4th order was almost independent of laterality. However, for different myopic levels, the individual aberrations of 2nd to 4th order with statistical differences obtained by the two devices were different.

**Table 2:** Z(2,0), Z(2,2), Z(3,1), Z(4,0) measured by the two devices within 6 subgroups

Zernike Coefficients (µm)	OD		OS	
	Pentacam	KR-1W	Pentacam	KR-1W
	Mean ± SD		Mean ± SD	
Subgroup 1–2	Mild myopia			
Z(2,0)	0.7093 ± 0.3199	-0.5287 ± 0.1765	0.8862 ± 0.3880	-0.5746 ± 0.2035
Z(2,2)	-0.8307 ± 0.4835	-0.6506 ± 0.4094	-0.9937 ± 0.5594	-0.7437 ± 0.4257
Z(3,1)	-0.0611 ± 0.0951	-0.1241 ± 0.0860	0.1334 ± 0.1353	0.1625 ± 0.1052
Z(4,0)	0.2191 ± 0.0845	0.2013 ± 0.0769	0.2245 ± 0.0693	0.1892 ± 0.0735
Subgroup 3–4	Moderate myopia			
Z(2,0)	0.6900 ± 0.4000	-0.6600 ± 0.4600	0.7600 ± 0.3900	-0.5000 ± 0.4500
Z(2,2)	-0.9900 ± 0.5100	-0.7500 ± 0.4100	-0.9800 ± 0.4700	-0.8100 ± 0.6400
Z(3,1)	-0.0720 ± 0.0880	-0.1000 ± 0.1100	0.1100 ± 0.1000	0.1500 ± 0.1100
Z(4,0)	0.2100 ± 0.0770	0.1700 ± 0.1800	0.2140 ± 0.0680^a^	0.2320 ± 0.2500^a^
Subgroup 5–6	Severe myopia			
Z(2,0)	0.7400 ± 0.3300	-0.6000 ± 0.3200	0.7900 ± 0.3600	-0.6800 ± 0.1700
Z(2,2)	-0.8200 ± 0.5300	-0.6800 ± 0.4800	-1.1000 ± 0.5100	-0.8500 ± 0.3800
Z(3,1)	-0.0270 ± 0.1100	-0.0700 ± 0.0890	0.0640 ± 0.0900	0.1000 ± 0.1100
Z(4,0)	0.2100 ± 0.0740	0.2000 ± 0.1400	0.2200 ± 0.0790	0.1700 ± 0.0950

All Zernike Coefficients mentioned above were statistically different (*P* < 0.05) between the two devices according to Paired t-tests or Wilcoxon two-related-samples tests

^a^ did not conform to the positive distribution, hence the numerical comparison should be assessed by the median (quartile): Pentacam: (0.233 (0.080)), KR-1W: (0.205 (0.056))

Regarding Pearson’s or Spearman’s correlation coefficients for both instruments, except for the vast majority of 5th and 6th order Zernike coefficients, the measurement of various parameters reached statistical differences. Generally, the correlation coefficients decreased or reached insignificance with an increased order of aberrations. Specifically, for 2nd order aberrations, the correlation coefficient of Z(2,0) was significantly smaller than that of Z(2, ± 2); for the aberrations most related to visual quality (3rd order HOAs and Z(4,0)), the correlation between the 2 devices was moderate; and for the other 4th order aberrations, low to moderate.

**Fig. 1:**
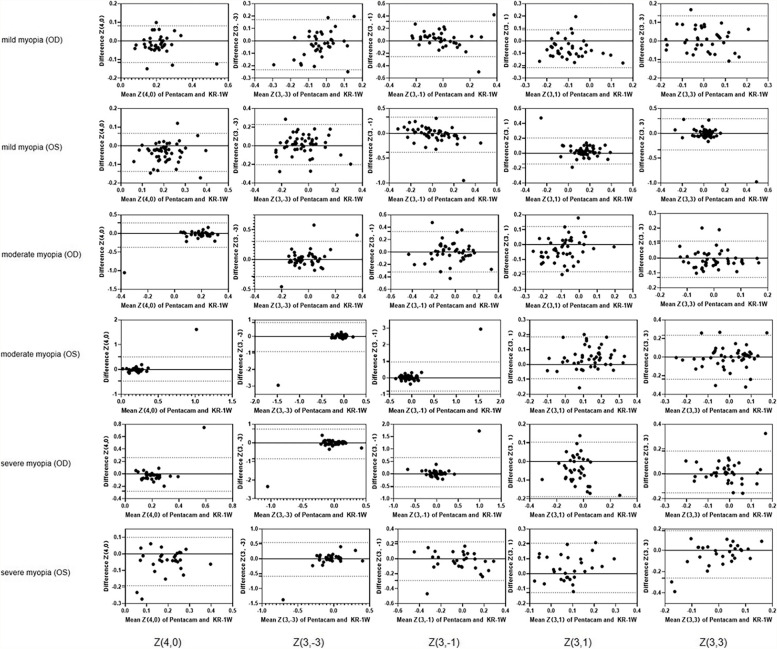
Bland—Altman plots of the values obtained from the Pentacam and the KR-1W and the corresponding 95% limits of agreement for the individual Zernike coefficients: Z(4,0), Z(3,-3), Z(3,-1), Z(3,1), Z(3,3)

In Bland–Altman analysis, bias values were close to zero in various parameters except for the second-order aberration, and the width of LoA for almost all aberrations was larger than 0.1 μm (supplemental Table 2). Furthermore, since primary SA and the third HOAs are the most critical factors influencing visual quality among all aberrations, special attention was paid to the agreement of Z(4,0), Z(3,1), Z(3,-1) (vertical coma), Z(3,3) (oblique trefoil), and Z(3,-3) (vertical trefoil) obtained by these two instruments. Figure 1 displays that almost all values are within the 95% LoA; however, the width of LoA was clinically too wide for all variables (width of LoA > 0.1 μm), indicating poor agreement in the measurement of aberrations between the two instruments [16].

## Discussion

A precise measurement of HOAs is a prerequisite for applying wavefront technology to clinical practice. The KR-1W and the Pentacam could provide vital information about the cornea and other ocular structures, such as pupillometry, keratometry, wavefront data, and topography data. Nowadays, the HOAs, one of the essential visual quality metrics, are gaining popularity. Previous studies have used various instruments for measuring HOAs in normal eyes [21–24]. Though, some studies have evaluated the agreement between Pentacam and KR-1W, focusing on the measurement of the basic ocular biometry and evaluating the agreement level in the relatively small sample size in normal people without exploring their agreement in measuring aberrations [25–30]. To the best of our knowledge, this is the first study elucidating the differences between the Pentacam and the KR-1W in measuring anterior corneal aberrations for myopes. As corneal refractive surgery is often performed on both eyes and knowledge about whether the agreement between the two aberrometers in corneal aberrations varies by the laterality is sparse, we further set eyes of a certain myopia degree into two subgroups according to the laterality.

In our study, we enrolled three kinds of subjects with different degrees of myopia: mild (-0,5D - -3.0D), moderate (-3.25D - -6.0D), and severe (≥ -6.25D). By observing subjects with varying myopia degrees, for the first time, we proved that these two devices could not be interchangeable when measuring all types of anterior corneal aberrations, regardless of myopia degree. Also, a previous study found a mirror relationship between the right and left eyes when measuring HOAs [31]. To elucidate the impact of the laterality of eyes on the agreements, we included both eyes, which were further grouped according to the degree of myopia. After pairwise evaluation of correlations and agreements of the aberrations measured by the two instruments within each subgroup, we found that the laterality of eyes had no impact on measurement agreement for either low-order aberrations or HOAs. We have, therefore, come to the quite comprehensive conclusion that the two devices are not interchangeable in the measurement of corneal aberrations, regardless of the level of myopia or the laterality of eyes.

When comparing whether there was a difference in the mean value of the individual aberrations obtained by the two devices, we likewise found a mirror relationship between the right and left eyes; namely, at the same degree of myopia, the types of aberration reaching differences were almost identical. In cases of severe myopia, for example, Z(2,0), Z(2,2), Z(3,1), and Z(4,0) measured by both devices were found to be different bilaterally (supplemental Table 1). In terms of the magnitude of the parameters among six subgroups, four certain corneal aberrations—(Z(2,0), Z(2,2), Z(3,1), Z(4,0))—between the instruments were significantly different. It seems to reflect a common trend in evaluating the mean values of aberration measurements with different ophthalmic instruments [32, 33]. A study, assessing the aberrations of four machines, revealed that the four machines differed in axial astigmatism, horizontal coma, and SA [32]. Also, another research, using a total of four devices to evaluate aberrations, found that the machines varied in the magnitude of defocus, axial astigmatism, trefoil, and SA [33]. Compared with the aforementioned two studies, our choice of the studied population (myopia population vs. normal population) and instruments are unique.

Additionally, the results for Pentacam were constantly larger than KR-1W for three anterior corneal aberrations (Z(2,0), Z(2,2), and Z(4,0)). Interestingly, two previous studies have also found that Pentacam overestimates some aberrations, with the values of Pentacam achieved 3.7 to 7 times larger than those of other machines (Pentacam vs. iTrace; Pentacam vs. Videokeratography) [20, 34]. Also, in terms of the values of the SA measurements, our results were around 0.2 μm, which is consistent with previously published studies [13, 34].

If one looks at the Bland-Altman graphs of the five parameters most relevant to visual quality, the majority of the measurement points lie between the upper LoA and lower LoA lines, with only few outside the LoAs. However, the width of the LoA, from the Bland-Altman analysis, is a more important metric to determine a good or poor agreement. Aberrations greater than 0.1 μm is considered clinically significant [16]. Taking all 3rd order aberrations and Z(4,0) as examples, the width of LoA intervals of Z(3,-1), Z(3,1), Z(3,-3), Z(3,3), and Z(4,0) were 0.5187–1.7645, 0.2812–0.3495, 0.4042–1.7457, 0.2437–0.6292 and 0.1969–0.9696, respectively. Obviously, the two machines in the study are not interchangeable in the measurement of corneal aberrations. Hence, the inherent characteristics of the two devices may contribute to the differences.

First, the wavelength of the light sources of the two devices may influence the results. A previous study, comparing the agreement of various aberrometers in measuring aberrations, revealed that the longitudinal chromatic aberrations caused by the different wavelengths used by various devices contributed to the measurement differences [22]. Actually, the wavelengths of the two instruments in the study varied (Pentacam: 475 nm blue light; KR-1W: 820-840 nm infrared light). Second, another reason for the discrepancy is the principle of the corneal aberration measurement. Pentacam uses Scheimpflug technology, which requires the acquisition of multiple corneal radial cross-sectional images for measuring corneal aberrations, based on which the cornea is reconstructed using a built-in algorithm. The KR-1W, which combines the Placido-disc topography and the Hartmann-Shack system, acquires anterior corneal aberrations by calculating computed topography data. Third, the Pentacam collects elevation information for up to 25,000 points, whereas KR-1W collects topography data for 13,680 points. We speculate that the difference in the amount of data may affect the final results. Furthermore, the subject's factors may influence the agreement between the two devices. Previous studies suggested that the tear film instability, and sudden eye movements can lead to errors of aberration measurement [17, 35]. Therefore, we asked the subjects to blink properly before each examination and instructed the participants to keep their gaze to provide a high-quality result. Finally, the difference in alignment may also account for the disagreement; the KR-1W’s center is fully automatic, whereas the Pentacam requires manual alignment.

There are also some limitations in our study. First, although measurements were taken immediately after a blink, we cannot guarantee that they were all taken precisely at the same time after blinking, especially between different subjects. Therefore, variability in the tear film could produce the observed variability during the measurement. Second, the qualities of the tear film could also influence corneal aberration measurements [35]. Though participants, diagnosed with dry eyes, were not included in the study, the ocular surface dryness was not quantitively measured.

In conclusion, the correlation between the two instruments was poor to moderate in terms of all 2nd to 6th order correlations, with the values of Pentacam significantly larger than KR-1W’s in three certain aberrations (Z(2,0), Z(2,2), Z(4,0)), regardless of the degree of myopia or the laterality of eyes. Furthermore, as the width of limits of agreement (LoA) between the two instruments was clinically too wide (> 0.1 μm) for almost all aberrations, the Pentacam and the KR-1W cannot be interchangeable for measuring anterior corneal aberrations in myopes.

## Supplementary Information

**Additional file 1: Supplemental Table 1. **Values of Zernike Coefficients and correlation coefficient.

**Additional file 2: Supplemental Table 2. **Mean differences and 95% Limits of Agreement(LoA) between the Pentacam and the KR-1W.

## Data Availability

The data of the study are available from the corresponding author upon reasonable request.

## Abbreviations

BCVA: Best corrected visual acuity
D: Diopter
HOAs: Higher-order aberrations
LoA: Limits of agreement
SD: Standard deviation
