# Supplementary material for: Comparison of anterior corneal aberrations measured by Scheimpflug and Placido Disc System for myopes
Source: BMC Ophthalmol. 2022 Dec 28;22:512. doi: 10.1186/s12886-022-02753-9 (PMC9798706; doi:10.1186/s12886-022-02753-9)
Supplement: Supplementary file 1 — Additional file 1: Supplemental Table 1. Values of Zernike Coefficients and correlation coefficient. [file 12886_2022_2753_MOESM1_ESM.docx]

| Supplemental Table 1.1: Values of Zernike Coefficients measured by Pentacam and KR-1W and correlation coefficient in mild myopia | | | | | | | | | | |
| --- | --- | --- | --- | --- | --- | --- | --- | --- | --- | --- |
| Subgroup 1 (mild myopia on the right eyes) | | | | | | Subgroup 2 (mild myopia on the left eyes) | | | | |
|  | Pentacam | | KR-1W | | Correlation Coefficient^#^ | Pentacam | | KR-1W | | Correlation Coefficient^#^ |
| Zernike Coefficients (µm) | Mean | SD | Mean | SD | R values | Mean | SD | Mean | SD | R values |
| Z (2,-2) | -0.0280^*^ | 0.3991^*^ | 0.0301^*^ | 0.3387^*^ | 0.949^+^ | -0.1621 | 0.3672 | -0.1199 | 0.2644 | 0.789^+^ |
| Z (2,0) | 0.7093^*^ | 0.3199^*^ | -0.5287^*^ | 0.1765^*^ | 0.510^+^ | 0.8862^*^ | 0.3880^*^ | -0.5746^*^ | 0.2035^*^ | 0.382^+^ |
| Z (2,2) | -0.8307^*^ | 0.4835^*^ | -0.6506^*^ | 0.4094^*^ | 0.958^+^ | -0.9937^*^ | 0.5594^*^ | -0.7437^*^ | 0.4257^*^ | 0.954^+^ |
| Z (3,-3) | -0.0201 | 0.0836 | -0.0510 | 0.1182 | 0.459^+^ | -0.0503 | 0.1331 | -0.0380 | 0.1317 | 0.653^+^ |
| Z (3,-1) | -0.0256 | 0.1717 | 0.0063 | 0.1487 | 0.739^+^ | 0.0306 | 0.2204 | 0.0029 | 0.1495 | 0.581^+^ |
| Z (3,1) | -0.0611^*^ | 0.0951^*^ | -0.1241^*^ | 0.0860^*^ | 0.635^+^ | 0.1334^*^ | 0.1353^*^ | 0.1625^*^ | 0.1052^*^ | 0.810^+^ |
| Z (3,3) | -0.0018 | 0.1016 | 0.0084 | 0.0945 | 0.793^+^ | 0.0274 | 0.1668 | 0.0067 | 0.0822 | 0.501^+^ |
| Z (4,-4) | 0.0463^*^ | 0.0614^*^ | 0.0085^*^ | 0.0569^*^ | 0.439^+^ | -0.0257^*^ | 0.0562^*^ | -0.0090^*^ | 0.0386^*^ | 0.453^+^ |
| Z (4,-2) | -0.0215 | 0.0359 | -0.0080 | 0.0413 | 0.527^+^ | 0.0116 | 0.0602 | 0.0117 | 0.0333 | 0.513^+^ |
| Z (4,0) | 0.2191^*^ | 0.0845^*^ | 0.2013^*^ | 0.0769^*^ | 0.703^+^ | 0.2245^*^ | 0.0693^*^ | 0.1892^*^ | 0.0735^*^ | 0.677^+^ |
| Z (4,2) | -0.0264 | 0.0610 | -0.0215 | 0.0706 | 0.620^+^ | -0.0250 | 0.0639 | -0.0179 | 0.0749 | 0.641^+^ |
| Z (4,4) | -0.0190^*^ | 0.0572^*^ | 0.0071^*^ | 0.0637^*^ | 0.334^+^ | -0.0372^*^ | 0.1437^*^ | 0.0029^*^ | 0.0733^*^ | 0.630^+^ |
| Z (5,-5) | -0.0108 | 0.0470 | 0.0019 | 0.0436 | 0.070 | -0.0133 | 0.0899 | 0.0000 | 0.0495 | 0.223^+^ |
| Z (5,-3) | -0.0033 | 0.0313 | -0.0003 | 0.0385 | 0.298 | -0.0069 | 0.0281 | -0.0039 | 0.0456 | 0.420^+^ |
| Z (5,-1) | 0.0126 | 0.0399 | 0.0016 | 0.0381 | 0.297 | 0.0048 | 0.0407 | -0.0037 | 0.0497 | 0.354^+^ |
| Z (5,1) | 0.0040^*^ | 0.0166^*^ | -0.0029^*^ | 0.0189^*^ | 0.589^+^ | 0.0015^*^ | 0.0209^*^ | 0.0082^*^ | 0.0135^*^ | 0.095 |
| Z (5,3) | 0.0009^*^ | 0.0194^*^ | -0.0086^*^ | 0.0296^*^ | 0.292 | 0.0077 | 0.0187 | 0.0055 | 0.0230 | 0.149 |
| Z (5,5) | 0.0030 | 0.0381 | 0.0152 | 0.0319 | 0.374^+^ | -0.0218^*^ | 0.0331^*^ | -0.0064^*^ | 0.0249^*^ | 0.016 |
| Z (6,-6) | -0.0023 | 0.0248 | 0.0050 | 0.0273 | 0.190 | 0.0052 | 0.0383 | 0.0050 | 0.0198 | 0.128 |
| Z (6,-4) | -0.0048 | 0.0139 | -0.0045 | 0.0175 | 0.204 | 0.0026 | 0.0160 | 0.0001 | 0.0161 | 0.236 |
| Z (6,-2) | -0.0034 | 0.0109 | 0.0001 | 0.0157 | 0.197 | 0.0075 | 0.0150 | 0.0021 | 0.0093 | -0.071 |
| Z (6,0) | 0.0069^*^ | 0.0216^*^ | -0.0066^*^ | 0.0274^*^ | 0.549^+^ | -0.0073 | 0.0188 | -0.0075 | 0.0218 | 0.316^+^ |
| Z (6,2) | 0.0100 | 0.0198 | 0.0052 | 0.0302 | 0.299 | 0.0103 | 0.0177 | 0.0034 | 0.0302 | 0.400^+^ |
| Z (6,4) | 0.0000 | 0.0198 | -0.0069 | 0.0284 | 0.202 | -0.0004 | 0.0267 | -0.0026 | 0.0263 | 0.160 |
| Z (6,6) | -0.0004 | 0.0346 | 0.0075 | 0.0304 | -0.268 | -0.0005 | 0.0315 | 0.0038 | 0.0286 | 0.011 |
| *: statistically significant measured by Paired t-tests or Wilcoxon two-related-samples tests, P < 0.05;  ^#^: correlation coefficient measured by Pearson or Spearman;  ^+^: statistically significant correlation, P < 0.05 | | | | | | | | | | |

| Supplemental Table 1.2: Values of Zernike Coefficients measured by Pentacam and KR-1W and correlation coefficient in moderate myopia | | | | | | | | | | |
| --- | --- | --- | --- | --- | --- | --- | --- | --- | --- | --- |
| Subgroup 3 (moderate myopia on the right eyes) | | | | | | Subgroup 4 (moderate myopia on the left eyes) | | | | |
|  | Pentacam | | KR-1W | | Correlation Coefficient^#^ | Pentacam | | KR-1W | | Correlation Coefficient^#^ |
| Zernike Coefficients (µm) | Mean | SD | Mean | SD | R values | Mean | SD | Mean | SD | R values |
| Z (2,-2) | -0.0002^*^ | 0.2800^*^ | 0.0350^*^ | 0.2300^*^ | 0.924^+^ | -0.0180^*^ | 0.3300^*^ | -0.0630^*^ | 0.2900^*^ | 0.940^+^ |
| Z (2,0) | 0.6900^*^ | 0.4000^*^ | -0.6600^*^ | 0.4600^*^ | 0.508^+^ | 0.7600^*^ | 0.3900^*^ | -0.5000^*^ | 0.4500^*^ | 0.250 |
| Z (2,2) | -0.9900^*^ | 0.5100^*^ | -0.7500^*^ | 0.4100^*^ | 0.926^+^ | -0.9800^*^ | 0.4700^*^ | -0.8100^*^ | 0.6400^*^ | 0.837^+^ |
| Z (3,-3) | -0.0210 | 0.0990 | -0.0120 | 0.1500 | 0.497^+^ | -0.0510 | 0.0960 | -0.0920 | 0.4400 | 0.526^+^ |
| Z (3,-1) | -0.0480 | 0.1700 | -0.0440 | 0.1700 | 0.534^+^ | -0.0800 | 0.1500 | -0.0025 | 0.4800 | 0.665^+^ |
| Z (3,1) | -0.0720^*^ | 0.0880^*^ | -0.1000^*^ | 0.1100^*^ | 0.699^+^ | 0.1100^*^ | 0.1000^*^ | 0.1500^*^ | 0.1100^*^ | 0.777^+^ |
| Z (3,3) | 0.0007 | 0.0790 | -0.0076 | 0.0760 | 0.649^+^ | -0.0020 | 0.1100 | -0.0039 | 0.1200 | 0.431^+^ |
| Z (4,-4) | 0.0410^*^ | 0.0620^*^ | 0.0270^*^ | 0.0760^*^ | 0.315^+^ | -0.0360^*^ | 0.0560^*^ | -0.0170^*^ | 0.0740^*^ | 0.140 |
| Z (4,-2) | -0.0200 | 0.0300 | -0.0130 | 0.0250 | 0.478^+^ | 0.0250 | 0.0430 | 0.0180 | 0.0430 | 0.486^+^ |
| Z (4,0) | 0.2100^*^ | 0.0770^*^ | 0.1700^*^ | 0.1800^*^ | 0.639^+^ | 0.2140^*^ | 0.0680^*^ | 0.2320^*^ | 0.2500^*^ | 0.564^+^ |
| Z (4,2) | -0.0100 | 0.0470 | -0.0054 | 0.1100 | 0.535^+^ | -0.0210 | 0.0700 | -0.0660 | 0.3300 | 0.607^+^ |
| Z (4,4) | -0.0160 | 0.0500 | -0.0066 | 0.0990 | 0.225 | -0.0023 | 0.0580 | 0.0440 | 0.3000 | 0.418^+^ |
| Z (5,-5) | -0.0073 | 0.0410 | -0.0003 | 0.0530 | 0.281 | -0.0130 | 0.0490 | 0.0200 | 0.1500 | 0.367^+^ |
| Z (5,-3) | 0.0010 | 0.0220 | 0.0060 | 0.0690 | -0.005 | -0.0029 | 0.0270 | -0.0280 | 0.1800 | 0.097 |
| Z (5,-1) | 0.0026 | 0.0350 | -0.0160 | 0.0930 | 0.209 | 0.0035 | 0.0410 | 0.0250 | 0.1900 | 0.385^+^ |
| Z (5,1) | 0.0019^*^ | 0.0160^*^ | -0.0130^*^ | 0.0450^*^ | 0.277 | 0.0043 | 0.0210 | 0.0065 | 0.0150 | 0.326^+^ |
| Z (5,3) | -0.0053 | 0.0160 | 0.0005 | 0.0160 | 0.053 | 0.0076 | 0.0210 | 0.0058 | 0.0470 | -0.062 |
| Z (5,5) | -0.0047 | 0.0380 | -0.0066 | 0.0930 | 0.156 | -0.0150 | 0.0430 | -0.0076 | 0.0600 | -0.202 |
| Z (6,-6) | 0.0008 | 0.0270 | -0.0037 | 0.0770 | -0.039 | -0.0049 | 0.0290 | -0.0020 | 0.0380 | 0.051 |
| Z (6,-4) | -0.0007 | 0.0160 | 0.0030 | 0.0270 | 0.054 | 0.0087 | 0.0200 | 0.0023 | 0.0300 | 0.059 |
| Z (6,-2) | -0.0050 | 0.0088 | -0.0047 | 0.0240 | -0.008 | 0.0009 | 0.0150 | 0.0003 | 0.0180 | 0.093 |
| Z (6,0) | 0.0059^*^ | 0.0270^*^ | -0.0069^*^ | 0.0260^*^ | -0.150 | -0.0005^*^ | 0.0250^*^ | 0.0035^*^ | 0.0660^*^ | 0.393^+^ |
| Z (6,2) | 0.0100 | 0.0200 | 0.0120 | 0.0580 | 0.035 | 0.0078 | 0.0260 | -0.0110 | 0.0930 | 0.157 |
| Z (6,4) | -0.0070 | 0.0200 | -0.0092 | 0.0420 | 0.203 | -0.0070 | 0.0200 | 0.0040 | 0.0780 | 0.157 |
| Z (6,6) | 0.0074 | 0.0310 | 0.0044 | 0.0210 | -0.089 | 0.0021 | 0.0190 | 0.0021 | 0.0620 | 0.016 |
| *: statistically significant measured by Paired t-tests or Wilcoxon two-related-samples tests, P < 0.05;  ^#^: correlation coefficient measured by Pearson or Spearman;  ^+^: statistically significant correlation, P < 0.05 | | | | | | | | | | |

| Supplemental Table 1.3: Values of Zernike Coefficients measured by Pentacam and KR-1W and correlation coefficient in severe myopia | | | | | | | | | | |
| --- | --- | --- | --- | --- | --- | --- | --- | --- | --- | --- |
| Subgroup 5 (severe myopia on the right eyes) | | | | | | Subgroup 6 (severe myopia on the left eyes) | | | | |
|  | Pentacam | | KR-1W | | Correlation Coefficient^#^ | Pentacam | | KR-1W | | Correlation Coefficient^#^ |
| Zernike Coefficients (µm) | Mean | SD | Mean | SD | R values | Mean | SD | Mean | SD | R values |
| Z (2,-2) | -0.0220 | 0.3800 | 0.0240 | 0.3100 | 0.928^+^ | 0.0400 | 0.3400 | 0.0051 | 0.2600 | 0.820^+^ |
| Z (2,0) | 0.7400^*^ | 0.3300^*^ | -0.6000^*^ | 0.3200^*^ | 0.098 | 0.7900^*^ | 0.3600^*^ | -0.6800^*^ | 0.1700^*^ | 0.517^+^ |
| Z (2,2) | -0.8200 ^*^ | 0.5300^*^ | -0.6800 ^*^ | 0.4800^*^ | 0.821^+^ | -1.1000^*^ | 0.5100^*^ | -0.8500^*^ | 0.3800^*^ | 0.889^+^ |
| Z (3,-3) | -0.0089 | 0.1600 | -0.0720 | 0.3900 | 0.547^+^ | -0.0410 | 0.1400 | -0.0630 | 0.3100 | 0.614^+^ |
| Z (3,-1) | -0.0250 | 0.2100 | 0.0420 | 0.3600 | 0.747^+^ | -0.0230 | 0.2100 | -0.0550 | 0.1900 | 0.785^+^ |
| Z (3,1) | -0.0270^*^ | 0.1100^*^ | -0.0700^*^ | 0.0890^*^ | 0.719^+^ | 0.0640^*^ | 0.0900^*^ | 0.1000^*^ | 0.1100^*^ | 0.665^+^ |
| Z (3,3) | -0.0240 | 0.0940 | -0.0084 | 0.0970 | 0.594^+^ | 0.0210 | 0.0740 | -0.0170 | 0.1200 | 0.421^+^ |
| Z (4,-4) | 0.0340 | 0.0620 | 0.0240 | 0.0620 | 0.570^+^ | -0.0260 | 0.0540 | -0.0430 | 0.1200 | 0.463^+^ |
| Z (4,-2) | -0.0220 | 0.0500 | -0.0250 | 0.0490 | 0.566^+^ | 0.0410 | 0.0430 | 0.0420 | 0.0550 | 0.436^+^ |
| Z (4,0) | 0.2100^*^ | 0.0740^*^ | 0.2000^*^ | 0.1400^*^ | 0.715^+^ | 0.2200^*^ | 0.0790^*^ | 0.1700^*^ | 0.0950^*^ | 0.644^+^ |
| Z (4,2) | -0.0170 | 0.0790 | -0.0420 | 0.2300 | 0.735^+^ | -0.0009 | 0.0830 | 0.0022 | 0.1100 | 0.601 |
| Z (4,4) | -0.0230 | 0.0830 | 0.0400 | 0.2900 | 0.471^+^ | -0.0120 | 0.0590 | 0.0310 | 0.3400 | 0.287 |
| Z (5,-5) | -0.0043 | 0.0370 | 0.0380 | 0.1800 | 0.127 | -0.0016 | 0.0340 | 0.0530 | 0.2800 | 0.153 |
| Z (5,-3) | 0.0001 | 0.0200 | -0.0270 | 0.1400 | 0.441^+^ | -0.0066 | 0.0380 | -0.0130 | 0.1100 | 0.511^+^ |
| Z (5,-1) | 0.0066 | 0.0540 | 0.0160 | 0.1200 | 0.718^+^ | 0.0042^*^ | 0.0450^*^ | -0.0250^*^ | 0.0630^*^ | 0.671^+^ |
| Z (5,1) | -0.0047 | 0.0200 | -0.0067 | 0.0170 | 0.405^+^ | 0.0070 | 0.0260 | 0.0120 | 0.0250 | 0.041 |
| Z (5,3) | -0.0003 | 0.0240 | 0.0038 | 0.0260 | 0.432^+^ | -0.0021 | 0.0230 | -0.0062 | 0.0490 | 0.027 |
| Z (5,5) | -0.0023 | 0.0350 | -0.0007 | 0.0490 | 0.238 | -0.0069 | 0.0410 | 0.0180 | 0.1100 | 0.324 |
| Z (6,-6) | 0.0046 | 0.0220 | -0.0002 | 0.0370 | -0.107 | -0.0068 | 0.0230 | 0.0075 | 0.0910 | 0.165 |
| Z (6,-4) | -0.0028 | 0.0130 | 0.0022 | 0.0190 | 0.123 | -0.0023 | 0.0180 | -0.0039 | 0.0530 | 0.081 |
| Z (6,-2) | -0.0006 | 0.0110 | -0.0020 | 0.0110 | 0.291 | -0.0003 | 0.0140 | 0.0023 | 0.0270 | -0.127 |
| Z (6,0) | 0.0018 | 0.0160 | 0.0008 | 0.0330 | 0.244^+^ | -0.0015 | 0.0230 | -0.0110 | 0.0310 | 0.271 |
| Z (6,2) | 0.0048^*^ | 0.0160^*^ | -0.0096^*^ | 0.0500^*^ | 0.382 | 0.0029 | 0.0160 | 0.0041 | 0.0420 | 0.343 |
| Z (6,4) | 0.0025 | 0.0200 | 0.0110 | 0.0720 | 0.216 | -0.0020 | 0.0210 | 0.0200 | 0.0910 | 0.269 |
| Z (6,6) | 0.0020 | 0.0210 | -0.0130 | 0.0890 | -0.288 | -0.0005 | 0.0300 | -0.0370 | 0.1800 | -0.147 |
| *: statistically significant measured by Paired t-tests or Wilcoxon two-related-samples tests, P < 0.05;  ^#^: correlation coefficient measured by Pearson or Spearman;  ^+^: statistically significant correlation, P < 0.05 | | | | | | | | | | |
