# Supplementary material for: Comparison of anterior corneal aberrations measured by Scheimpflug and Placido Disc System for myopes
Source: BMC Ophthalmol. 2022 Dec 28;22:512. doi: 10.1186/s12886-022-02753-9 (PMC9798706; doi:10.1186/s12886-022-02753-9)
Supplement: Supplementary file 2 — Additional file 2: Supplemental Table 2. Mean differences and 95% Limits of Agreement(LoA) between the Pentacam and the KR-1W. [file 12886_2022_2753_MOESM2_ESM.docx]

| Supplemental Table 2.1 Mean differences and 95% Limits of Agreement (LoA) between the two devices in mild myopia | | | | | | | | | |
| --- | --- | --- | --- | --- | --- | --- | --- | --- | --- |
|  | Subgroup 1 (mild myopia on the right eyes) | | | | | Subgroup 2 (mild myopia on the left eyes) | | | |
| Zernike Coefficients | Mean±SD | | Lower LoA | Upper LoA | Width of LoA | Mean±SD | Lower LoA | Upper LoA | Width of LoA |
| Z (2,-2) | 0.0581±0.1324 | | -0.2014 | 0.3176 | 0.5190 | 0.0422±0.2269 | -0.4025 | 0.4870 | 0.8895 |
| Z (2,0) | -1.2380±0.2754 | | -1.7780 | -0.6983 | 2.4763 | -1.4610±0.3540 | -2.1550 | -0.7670 | 2.9220 |
| Z (2,2) | 0.1801±0.1481 | | -0.1102 | 0.4704 | 0.5806 | 0.2500±0.2000 | -0.1420 | 0.6420 | 0.7840 |
| Z (3,-3) | -0.0309±0.1031 | | -0.2330 | 0.1712 | 0.4042 | 0.0123±0.1102 | -0.2038 | 0.2283 | 0.4321 |
| Z (3,-1) | 0.0319±0.1454 | | -0.2531 | 0.3168 | 0.5699 | -0.0277±0.1806 | -0.3817 | 0.3263 | 0.7080 |
| Z (3,1) | -0.0630±0.0778 | | -0.2156 | 0.0896 | 0.3052 | 0.0291±0.0891 | -0.1457 | 0.2038 | 0.3495 |
| Z (3,3) | 0.0102±0.0634 | | -0.1142 | 0.1345 | 0.2487 | -0.0207±0.1605 | -0.3353 | 0.2939 | 0.6292 |
| Z (4,-4) | -0.0378±0.0701 | | -0.1752 | 0.0995 | 0.2747 | 0.0166±0.0565 | -0.0941 | 0.1274 | 0.2215 |
| Z (4,-2) | 0.0136±0.0423 | | -0.0693 | 0.0965 | 0.1658 | 0.0001±0.0476 | -0.0932 | 0.0934 | 0.1866 |
| Z (4,0) | -0.0178±0.0502 | | -0.1163 | 0.0806 | 0.1969 | -0.0353±0.0523 | -0.1377 | 0.0671 | 0.2048 |
| Z (4,2) | 0.0049±0.0613 | | -0.1152 | 0.1250 | 0.2402 | 0.0071±0.0649 | -0.1202 | 0.1343 | 0.2545 |
| Z (4,4) | 0.0262±0.0700 | | -0.1111 | 0.1634 | 0.2745 | 0.0401±0.1317 | -0.2180 | 0.2982 | 0.5162 |
| Z (5,-5) | 0.0127±0.0618 | | -0.1085 | 0.1338 | 0.2423 | 0.0133±0.0880 | -0.1592 | 0.1859 | 0.3451 |
| Z (5,-3) | 0.0031±0.0417 | | -0.0787 | 0.0849 | 0.1636 | 0.0030±0.0424 | -0.0801 | 0.0861 | 0.1662 |
| Z (5,-1) | -0.0111±0.0463 | | -0.1017 | 0.0796 | 0.1813 | -0.0085±0.0519 | -0.1102 | 0.0931 | 0.2033 |
| Z (5,1) | -0.0069±0.0162 | | -0.0386 | 0.0248 | 0.0634 | 0.0067±0.0259 | -0.0441 | 0.0575 | 0.1016 |
| Z (5,3) | -0.0095±0.0324 | | -0.0730 | 0.0540 | 0.1270 | -0.0021±0.0259 | -0.0529 | 0.0487 | 0.1016 |
| Z (5,5) | 0.0122±0.0376 | | -0.0615 | 0.0859 | 0.1474 | 0.0154±0.0411 | -0.0651 | 0.0959 | 0.1610 |
| Z (6,-6) | 0.0073±0.0357 | | -0.0626 | 0.0772 | 0.1398 | -0.0002±0.0371 | -0.0729 | 0.0724 | 0.1453 |
| Z (6,-4) | 0.0003±0.0202 | | -0.0394 | 0.0399 | 0.0793 | -0.0025±0.0199 | -0.0415 | 0.0364 | 0.0779 |
| Z (6,-2) | 0.0035±0.0185 | | -0.0327 | 0.0397 | 0.0724 | -0.0055±0.0190 | -0.0427 | 0.0317 | 0.0744 |
| Z (6,0) | -0.0134±0.0238 | | -0.0601 | 0.0333 | 0.0934 | -0.0002±0.0239 | -0.0470 | 0.0467 | 0.0937 |
| Z (6,2) | -0.0048±0.0307 | | -0.0650 | 0.0554 | 0.1204 | -0.0069±0.0287 | -0.0631 | 0.0493 | 0.1124 |
| Z (6,4) | -0.0068±0.0316 | | -0.0688 | 0.0551 | 0.1239 | -0.0022±0.0375 | -0.0758 | 0.0714 | 0.1472 |
| Z (6,6) | 0.0079±0.0431 | | -0.0766 | 0.0924 | 0.1690 | 0.0043±0.0419 | -0.0779 | 0.0865 | 0.1644 |
| Supplemental Table 2.2 Mean differences and 95% Limits of Agreement (LoA) between the two devices in moderate myopia | | | | | | | | | |
|  | Subgroup 3 (moderate myopia on the right eyes) | | | | | Subgroup 4 (moderate myopia on the left eyes) | | | |
| Zernike Coefficients | Mean±SD | Lower LoA | | Upper LoA | Width of LoA | Mean±SD | Lower LoA | Upper LoA | Width of LoA |
| Z (2,-2) | 0.0349±0.1137 | -0.1878 | | 0.2577 | 0.4455 | -0.0451±0.1137 | -0.2679 | 0.1777 | 0.4456 |
| Z (2,0) | -1.3520±0.5509 | -2.4320 | | -0.2722 | 2.7042 | -1.2650±0.4632 | -2.1720 | -0.3569 | 2.5289 |
| Z (2,2) | 0.2378±0.2008 | -0.1558 | | 0.6313 | 0.7871 | 0.1752±0.5762 | -0.9542 | 1.3040 | 2.2582 |
| Z (3,-3) | 0.0092±0.1508 | -0.2863 | | 0.3047 | 0.5910 | -0.0406±0.4453 | -0.9134 | 0.8323 | 1.7457 |
| Z (3,-1) | 0.0038±0.1662 | -0.3219 | | 0.3295 | 0.6514 | 0.0775±0.4501 | -0.8048 | 0.9597 | 1.7645 |
| Z (3,1) | -0.0307±0.0776 | -0.1827 | | 0.1213 | 0.3040 | 0.0450±0.0717 | -0.0956 | 0.1856 | 0.2812 |
| Z (3,3) | -0.0083±0.0622 | -0.1302 | | 0.1135 | 0.2437 | -0.0018±0.1205 | -0.2379 | 0.2343 | 0.4722 |
| Z (4,-4) | -0.0138±0.1112 | -0.2317 | | 0.2041 | 0.4358 | 0.0196±0.0866 | -0.1501 | 0.1893 | 0.3394 |
| Z (4,-2) | 0.0070±0.0244 | -0.0408 | | 0.0548 | 0.0956 | -0.0070±0.0495 | -0.1041 | 0.0901 | 0.1942 |
| Z (4,0) | -0.0452±0.1670 | -0.3725 | | 0.2821 | 0.6546 | 0.0188±0.2473 | -0.4660 | 0.5036 | 0.9696 |
| Z (4,2) | 0.0046±0.1055 | -0.2023 | | 0.2115 | 0.4138 | -0.0455±0.3414 | -0.7146 | 0.6236 | 1.3382 |
| Z (4,4) | 0.0093±0.1043 | -0.1951 | | 0.2137 | 0.4088 | 0.0462±0.3066 | -0.5548 | 0.6472 | 1.2020 |
| Z (5,-5) | 0.0070±0.0591 | -0.1089 | | 0.1228 | 0.2317 | 0.0332±0.1652 | -0.2906 | 0.3570 | 0.6476 |
| Z (5,-3) | 0.0050±0.0752 | -0.1424 | | 0.1525 | 0.2949 | -0.0248±0.1950 | -0.4070 | 0.3574 | 0.7644 |
| Z (5,-1) | -0.0190±0.0990 | -0.2131 | | 0.1750 | 0.3881 | 0.0213±0.2171 | -0.4042 | 0.4468 | 0.8510 |
| Z (5,1) | -0.0145±0.0453 | -0.1032 | | 0.0743 | 0.1775 | 0.0021±0.0235 | -0.0440 | 0.0482 | 0.0922 |
| Z (5,3) | 0.0058±0.0221 | -0.0375 | | 0.0491 | 0.0866 | -0.0017±0.0545 | -0.1086 | 0.1051 | 0.2137 |
| Z (5,5) | -0.0019±0.1019 | -0.2016 | | 0.1978 | 0.3994 | 0.0078±0.0850 | -0.1588 | 0.1744 | 0.3332 |
| Z (6,-6) | -0.0044±0.0769 | -0.1552 | | 0.1464 | 0.3016 | 0.0030±0.0502 | -0.0955 | 0.1014 | 0.1969 |
| Z (6,-4) | 0.0037±0.0268 | -0.0488 | | 0.0562 | 0.1050 | -0.0064±0.0402 | -0.0852 | 0.0723 | 0.1575 |
| Z (6,-2) | 0.0003±0.0257 | -0.0501 | | 0.0508 | 0.1009 | -0.0006±0.0235 | -0.0467 | 0.0455 | 0.0922 |
| Z (6,0) | -0.0128±0.0428 | -0.0966 | | 0.0711 | 0.1677 | 0.0040±0.0775 | -0.1480 | 0.1559 | 0.3039 |
| Z (6,2) | 0.0021±0.0577 | -0.1111 | | 0.1152 | 0.2263 | -0.0190±0.1038 | -0.2225 | 0.1844 | 0.4069 |
| Z (6,4) | -0.0022±0.0445 | -0.0895 | | 0.0851 | 0.1746 | 0.0109±0.0874 | -0.1603 | 0.1822 | 0.3425 |
| Z (6,6) | -0.0030±0.0391 | -0.0796 | | 0.0735 | 0.1531 | 0.0000±0.0677 | -0.1328 | 0.1327 | 0.2655 |

| Supplemental Table 2.3 Mean differences and 95% Limits of Agreement (LoA) between the two devices in severe myopia | | | | | | | | |
| --- | --- | --- | --- | --- | --- | --- | --- | --- |
|  | Subgroup 5 (severe myopia on the right eyes) | | | | Subgroup 6 (severe myopia on the left eyes) | | | |
| Zernike Coefficients | Mean±SD | Lower LoA | Upper LoA | Width of LoA | Mean±SD | Lower LoA | Upper LoA | Width of LoA |
| Z (2,-2) | 0.0462±0.1484 | -0.2447 | 0.3371 | 0.5818 | -0.0353±0.1946 | -0.4167 | 0.3460 | 0.7627 |
| Z (2,0) | -1.3430±0.4720 | -2.2680 | -0.4179 | 2.6859 | -1.4700±0.3043 | -2.0670 | -0.8737 | 2.9407 |
| Z (2,2) | 0.1371±0.3070 | -0.4645 | 0.7388 | 1.2033 | 0.2481±0.2461 | -0.2342 | 0.7304 | 0.9646 |
| Z (3,-3) | -0.0627±0.4063 | -0.8589 | 0.7336 | 1.5925 | -0.0212±0.2888 | -0.5873 | 0.5448 | 1.1321 |
| Z (3,-1) | 0.0667±0.3013 | -0.5238 | 0.6572 | 1.1810 | -0.0320±0.1323 | -0.2913 | 0.2274 | 0.5187 |
| Z (3,1) | -0.0427±0.0747 | -0.1892 | 0.1037 | 0.2929 | 0.0385±0.0846 | -0.1273 | 0.2042 | 0.3315 |
| Z (3,3) | 0.0152±0.0860 | -0.1534 | 0.1837 | 0.3371 | -0.0384±0.1134 | -0.2607 | 0.1840 | 0.4447 |
| Z (4,-4) | -0.0092±0.0639 | -0.1344 | 0.1160 | 0.2504 | -0.0163±0.1082 | -0.2284 | 0.1957 | 0.4241 |
| Z (4,-2) | -0.0030±0.0297 | -0.0611 | 0.0552 | 0.1163 | 0.0009±0.0454 | -0.0882 | 0.0899 | 0.1781 |
| Z (4,0) | -0.0094±0.1384 | -0.2807 | 0.2619 | 0.5426 | -0.0465±0.0746 | -0.1927 | 0.0997 | 0.2924 |
| Z (4,2) | -0.0242±0.2206 | -0.4566 | 0.4081 | 0.8647 | 0.0031±0.0903 | -0.1738 | 0.1800 | 0.3538 |
| Z (4,4) | 0.0626±0.2986 | -0.5228 | 0.6479 | 1.1707 | 0.0430±0.3207 | -0.5855 | 0.6715 | 1.2570 |
| Z (5,-5) | 0.0422±0.1825 | -0.3155 | 0.3998 | 0.7153 | 0.0546±0.2799 | -0.4941 | 0.6032 | 1.0973 |
| Z (5,-3) | -0.0270±0.1380 | -0.2975 | 0.2436 | 0.5411 | -0.0066±0.1013 | -0.2051 | 0.1920 | 0.3971 |
| Z (5,-1) | 0.0097±0.1016 | -0.1894 | 0.2088 | 0.3982 | -0.0287±0.0570 | -0.1403 | 0.0829 | 0.2232 |
| Z (5,1) | -0.0020±0.0166 | -0.0346 | 0.0306 | 0.0652 | 0.0054±0.0293 | -0.0520 | 0.0628 | 0.1148 |
| Z (5,3) | 0.0041±0.0266 | -0.0481 | 0.0562 | 0.1043 | -0.0040±0.0597 | -0.1210 | 0.1130 | 0.2340 |
| Z (5,5) | 0.0016±0.0557 | -0.1076 | 0.1108 | 0.2184 | 0.0246±0.1176 | -0.2060 | 0.2551 | 0.4611 |
| Z (6,-6) | -0.0049±0.0420 | -0.0872 | 0.0774 | 0.1646 | 0.0143±0.0987 | -0.1791 | 0.2077 | 0.3868 |
| Z (6,-4) | 0.0049±0.0221 | -0.0384 | 0.0483 | 0.0867 | -0.0016±0.0608 | -0.1207 | 0.1175 | 0.2382 |
| Z (6,-2) | -0.0014±0.0128 | -0.0265 | 0.0237 | 0.0502 | 0.0027±0.0314 | -0.0588 | 0.0641 | 0.1229 |
| Z (6,0) | -0.0010±0.0336 | -0.0669 | 0.0649 | 0.1318 | -0.0094±0.0338 | -0.0757 | 0.0569 | 0.1326 |
| Z (6,2) | -0.0144±0.0501 | -0.1127 | 0.0839 | 0.1966 | 0.0012±0.0340 | -0.0655 | 0.0679 | 0.1334 |
| Z (6,4) | 0.0086±0.0738 | -0.1360 | 0.1532 | 0.2892 | 0.0217±0.0887 | -0.1522 | 0.1956 | 0.3478 |
| Z (6,6) | -0.0154±0.0956 | -0.2027 | 0.1720 | 0.3747 | -0.0360±0.1822 | -0.3932 | 0.3212 | 0.7144 |
